# Association of traumatic events with levels of psychological distress and depressive symptoms in male asylum seekers and refugees resettled in Italy

**DOI:** 10.1186/s12888-020-02988-0

**Published:** 2020-12-01

**Authors:** Michela Nosè, Lorenzo Tarsitani, Federico Tedeschi, Claudia Lotito, Paola Massetti, Marianna Purgato, Valentina Roselli, Liliana Todini, Giulia Turrini, Corrado Barbui

**Affiliations:** 1 WHO Collaborating Centre for Research and Training in Mental Health and Service Evaluation, Department of Neuroscience, Biomedicine and Movement Sciences, Section of Psychiatry, University of Verona, Verona, Italy; 2 Department of Neurosciences and Mental Health, Policlinico Umberto I, Sapienza University of Rome, Rome, Italy; 3 Department of Public Health and Infectious Diseases, Sapienza University of Rome, Rome, Italy

**Keywords:** Refugee, Trauma, Psychological distress, Mental health

## Abstract

**Background:**

In recent years there has been a progressive rise in the number of asylum seekers and refugees displaced from their country of origin, with significant social, economic, public health and mental health implications. The aim of this study is to (1) describe the level of psychological distress and frequency of psychiatric disorders in a sample of male asylum seekers and refugees across different ethnic groups resettled in Italy; (2) establish whether the number of traumatic events experienced before, during and after the migration process is associated with level of psychological distress and depressive symptoms.

**Methods:**

In two large Italian catchment areas, over a period of 1 year a consecutive series of male asylum seekers and refugees, aged 18 or above and included in the Italian protection system, were screened for psychological distress and psychiatric disorders using validated questionnaires.

**Results:**

During the study period, 252 male asylum seekers or refugees were recruited. More than one-third of the participants (34.5%) showed clinically relevant psychological distress, and one-fourth (22.2%), met the criteria for a psychiatric diagnosis, mainly Post Traumatic Stress Disorder and depressive disorders. The number of traumatic events turned out to be a risk factor for both clinically relevant psychological distress and depressive disorders. Receiving good social support emerged as a protective factor, while migrants with unclear status were at higher risk of psychological distress than those holding or awaiting a permission.

**Discussion:**

In an unselected sample of male asylum seekers and refugees, after around 1 year of resettlement in Italy, the frequency of psychological distress and psychiatric disorders was substantial and clinically relevant. The association between traumatic events, especially post-migration problems, and mental health conditions suggests the need of developing services to assist refugees and asylum seekers to address the multi-faceted problems they experience, such as social support in host country, legal problems concerning permit status and asylum procedure, and family reunification, as well as addressing trauma and mental health issues.

**Supplementary Information:**

The online version contains supplementary material available at 10.1186/s12888-020-02988-0.

## Background

In recent years there has been a progressive rise in the number of asylum seekers and refugees displaced from their country of origin and hosted in high income countries, with significant social, economic, humanitarian and public health implications [1, 2]. Of particular concern is the mental health of this population. As compared with the general population, asylum seekers and refugees experience higher rates of psychological distress and mental health disorders, including depression, anxiety, somatoform disorders, disorders specifically related to stress and severe mental disorders [3, 4]. Although a recent umbrella review of epidemiological studies highlighted substantial variability in prevalence rates, depression and anxiety were at least as frequent as post-traumatic stress disorder (PTSD), accounting for up to 40% of asylum seekers and refugees [5]. As most studies were on post-traumatic stress disorder, the review called for more studies on depression, anxiety and other mental health conditions.

High rates of psychological distress and mental health disorders may be related to exposure to traumatic events in the country of origin and during the migration process. After displacement, post-migration stressors, such as resettlement, perceived stigma, discrimination, social insecurity and uncertainty about the future, may represent additional risk factors for the development of psychological distress and mental health conditions [6, 7]. Overall, the number of trauma events may represent a key factor. Georgiadou and colleagues [8], in a sample of Syrian refugees resettled in Germany, showed that severe PTSD symptoms were associated with higher number of traumatic events, and similar findings were found by Tinghög and colleagues [9] among Syrian refugees resettled in Sweden. In a sample of women across several ethnic groups resettled in Australia, Schweitzer and colleagues [10] showed that higher numbers of traumatic events and post-migration living difficulties predicted higher depression and somatic symptoms. At the moment evidence on this association exists for Syrian refugees or women across different ethnic groups. As men and women may be exposed to different trauma experiences with gender-specific characteristics, and may also react differently to similar stressors, it remains uncertain whether this association holds true for populations of male refugees across different ethnic groups [11]. Against this background, the current study sought to (1) describe the level of psychological distress and frequency of mental health conditions in a sample of male asylum seekers and refugees across different ethnic groups resettled in Italy; (2) establish whether the number of traumatic events experienced before, during and after the migration process is associated with the levels of psychological distress and depressive symptoms.

## Methods

The methods employed in this study were described in our previous study [12] and are briefly reported. In this observational study, we followed the STROBE guidelines.

### Study setting and participant enrolment

In Italy, the protection system for asylum seekers and refugees (SPRAR system) was established by Law No 189/2002 and is made up of a network of local institutions that implement reception projects for asylum seekers. Local institutions, in cooperation with voluntary sector organisations, undertake integrated reception interventions that include food, housing, legal and social guidance and support, access to health care services, and the development of individual programmes to promote socioeconomic inclusion and integration.

Participants were recruited in Verona among asylum seekers and refugees seeking legal support from the Italian Council of Refugees of Verona, and in Rome among asylum seekers and refugees seeking health care support (routine screening of infectious diseases) from the Day Service of Migration Medicine of the Umberto I University Hospital in Rome. Both settings are organized to provide care to male refugees only. In both settings, during a 12-month recruitment period, asylum seekers and refugees seeking support were consecutively offered to participate. Participants aged 18 or older were included if they gave informed written consent. No formal exclusion criteria were applied. Before being enrolled in the study, participants were informed about the nature and scope of the study in a form understandable to them. Participants were advised that their decision about participation would have no impact on their visa status or their family’s visa status. This ensured that participation was completely voluntary and prevented biased responding – either exaggerating or minimising problems in the belief that this may help them obtain a permanent visa. Subsequent assessment interviews were arranged to best suit the participant – either immediately, at their next scheduled face-to-face appointment, or at a separate face-to-face appointment. When required, cultural mediators were involved during the interview process, supporting the participants in difficulties related to language or in helping the researchers to conduct the interviews in a culturally appropriate manner. Participants with mental disorders or clinically significant symptoms were offered referrals to community mental health services as appropriate. Some participants of the present study were also included in our previous study [12]. In this study recruitment was conducted in two different Italian areas to get a more representative sample. Moreover, the analysis on this sample are different from previous work, focusing on trauma events, the relationship between traumatic events and psychological distress, depressive symptoms and PTSD, and gender impact.

The study was conducted in compliance with the Helsinki Declaration and received Ethics Committee approvals from the University of Verona and the Sapienza University of Rome.

### Training of the cultural-linguistic mediators

Cultural-linguistic mediators involved in the project were specifically trained in working with asylum seekers. The training course was offered by the project staff to ensure clear communication among all actors dealing with persons who left their country of origin and formally applied for asylum in another country. Focus was placed on interview techniques, instruments and different types of interviews, so that cultural-linguistic mediators were skilled in all settings and were able to adequately assist the interdisciplinary team in detecting psychological needs and assisting the persons in a culturally appropriate manner. All cultural-linguistic mediators followed a code of conduct, complying with the principles of linguistic accuracy and neutrality, impartiality, confidentiality, demeanour, and avoiding activities that might lead to a conflict of interests. Cultural mediators were briefed on the aims of the study, study procedures, and ethical obligations (e.g. confidentiality, right to refuse to answer questions) and were required to formally declare that they have truthfully and faithfully translated the explanation of the research project to the best of their skills and ability.

### Assessment tools

Socio-demographic and clinical characteristics, including age, country of birth, social background, religion, pre-migration factors, migration history and post-migration factors were collected using an ad-hoc form. Perceived social support in the host country was rated during the interview on a 5-point Likert scale ranging from very poor to very good. The Life Events Checklist (LEC) was used to assess exposure to potentially traumatic events on a 5-point nominal scale. The Life Events Checklist was developed at the National Center for Posttraumatic Stress Disorder (PTSD) concurrently with the Clinician Administered PTSD Scale (CAPS) to facilitate the diagnosis of PTSD [13]. In order to ascertain the presence and severity of psychological distress the General Health Questionnaire-12 items (GHQ-12) was used. The GHQ-12 is a self-report questionnaire which assesses the situation over the past few weeks and is used for identifying individuals with general psychological distress and to screen for common mental disorders. The items are scored on a 4-point severity scale ranging from 1 to 4. We used the recommended and more usual scoring (0–0–1 − 1) [14] and created an overall score by summing the scores of all 12 items, with higher scores indicating worse conditions. A cut-off of 4 or more points was considered as an indication of psychological distress. Study participants scoring positive to the GHQ-12 (≥4) were further assessed for the presence of a psychiatric diagnosis using the MINI-International Neuropsychiatric Interview (M.I.N.I.), a brief structured interview for the major Axis I psychiatric disorders in DSM-IV and ICD-10. Validation and reliability studies have been done comparing the M.I.N.I. to the Structured Clinical Interview- Patient version (SCID-P) and the Composite International Diagnostic Interview (CIDI). The results of these studies show that the M.I.N.I. has acceptably high validation and reliability scores, but can be administered in a much shorter period of time than the above referenced instruments [15]. In addition, study participants scoring positive to the GHQ-12 were also assessed for the presence of clinically relevant depressive symptoms using the Hamilton Rating Scale for Depression (HRSD), a clinician-administered depression assessment scale designed for adults, aimed at rating severity of depression. With HRSD total score, a four-group variable was created: “no depressive symptoms” for scores up to 7, “mild depressive symptoms” for scores between 8 and 17, “moderate depressive symptoms” for scores between 18 and 24 and “severe depressive symptoms” for scores of 25 or more [16]. The different variables of the scale are measured either on five-point or three-point scales and the total score for the patient is obtained by summing the scores of variables.

### Statistical analysis

Means and standard deviations (in case of continuous variables) and absolute numbers and percentages (in case of categorical variables) were calculated for socio-demographic characteristics (age, sex, marital status, having at least one child, years of education and educational level, country of origin, religion), pre-migration characteristics (occupational status, reason for migration, family agreement for migration, living status), migration characteristics (time from departure to first visit, route of arrival, detention or not, level of social support), post-migration characteristics (length of stay in Italy at the time of first visit and legal status) and clinical characteristics (number of traumatic events as measured by LEC, psychological distress as measured by GHQ-12, psychological diagnosis as measured by M.I.N.I., both as a percentage of the total sample and of those with psychological distress only). Confidence intervals were calculated for the percentage of migrants with psychological distress and, among those, for the percentage of those with a psychiatric diagnosis.

As first step, the percentage of people with psychological distress was calculated for asylum seekers and refugees with respectively a low (2 or 3), median (4 or 5) or high (at least 6) number of traumatic events, as measured by the LEC scale, together with its confidence interval. Test of linear trend of log-odds was performed. The existence of a monotonic trend between LEC (grouped as above) and the HRSD score was investigated through the Jonckheere-Terpstra test [17]. Moreover, the association of LEC with GHQ and HRSD was assessed also by using them as interval-level variables, through calculation of Spearman’s correlation.

As second step psychological distress (GHQ-12) and presence of a diagnosis as measured by M.I.N.I. were used as dependent variables in a sequential logit model [17], i.e. a logit regression was performed on presence of psychological distress as measured by GHQ-12 and, among those scoring positive, a further logit regression was implemented, having presence of a M.I.N.I. diagnosis as outcome. Furthermore, severity of depression (HRSD) was used as dependent variable in a regression. Given the skewness to the right of such variable, we used its squared-root transformation as an outcome in order to have approximately normally distributed regression residuals. The following independent variables were used in the regression analyses: number of traumatic events (as measured by the LEC scale), educational level (dichotomized as “At least High School” vs “At most Junior High School”), reason for migration (“Terroristic threat” vs “Other”), presence of good social support (“Yes” in case the choice in a 5-point Likert scale was “Good” or “Very good”, “No” otherwise), permit to stay in the country (“Denied”, “Granted or awaiting” and “Unclear”), marital status (“Married or cohabiting” vs “Other”), age at the first visit and time from departure to first visit (expressed in years, as continuous variables), citizenship (divided into three groups: “Africa”, “Pakistan” and “Other”). In both models, the global significance of regressions was assessed first, before analyzing each predictor separately.

We performed two sensitivity analyses. First, the GHQ logistic regression was repeated using the Verona sample only. Then, we considered the Hamilton score as a four-group variable in ordinal logit model.

## Results

### Socio-demographic characteristics

During the study period, 252 male asylum seekers or refugees were invited to participate. All participants agreed to be involved. Table 1 summarizes the socio-demographic and clinical characteristics of the sample. The mean age of participants was 29 years. Almost half of the participants were Pakistani citizens (48%), and slightly more than one third were African citizens. Most participants were of Muslim religion (83%).

**Table 1:** Socio-demographic and migration characteristics of the included participants (*N* = 252)

Variable	n	% or mean (SD)
Socio-demographic characteristics		
**Age, years (mean, SD)**		28.7 (7.3)
**Sex, male**	252	100.0
**Marital status**		
Married	65	25.8
Single/other	187	74.2
**Having children**		
Yes	65	26.0
No	185	74.0
**Education, years (mean, SD)**		8.0 (5.1)
**Education level**		
Illiterate	45	18.0
Primary School/ junior high school	122	48.8
High School	60	24.0
University degree and above	23	9.2
**Country of Origin**		
Pakistan	121	48.0
Africa	87	34.5
other	44	17.5
**Religion**		
Muslim	201	82.7
Other	42	17.3
Pre- Migration characteristics		
**Occupational status**		
Unemployed	34	13.9
Employed	199	81.2
Students	12	4.9
**Reason for migration**		
Terroristic threats	60	23.8
Family/Community problems	47	18.7
Political problems	31	12.3
Religious problems	17	6.7
Other threats	37	14.7
Other	13	5.2
Unknown data	47	18.7
**Family’s agreement for departure**	172	72.0
**Living conditions**		
Alone	12	4.8
With partner (+/−children)	57	23.0
With parents	143	57.7
Other	36	14.5
Migration characteristics		
**Time after departures, years (mean, SD)**		3.3 (3.2)
**Route of arrival**		
Balkan	108	42.9
Easter Mediterranean	27	10.7
African Mediterranean	83	32.9
Other	34	13.5
Post-Migration characteristics		
**Detention during migration**	88	37.4
**Length of stay in Italy, months (mean, SD)**		14.1 (22.9)
**Legal status**		
Subsidiary permission	30	11.9
Humanitarian permission	35	13.9
Refugee status	44	17.5
Rejected request	106	42.1
Dublin Convention	8	3.2
Awaiting	17	6.7
Unknown data	12	4.8
**Social support**		
At least good	171	67.9
Fair	61	24.2
At most poor	20	7.9

Around one fourth had a partner, and around half received primary or lower secondary education, with 9% of study participants holding a University degree.

In terms of pre-migration characteristics, Table 1 reports data related to occupational status, living conditions and reasons for migration. With respect to migration characteristics, 43% arrived through the Balkan route, 37% had undergone detention, and 68% deemed the social support received during migration as good or very good. Looking at post-migration characteristics, the mean time after departure was around 3 years and the mean length of stay in Italy at first visit was 14.1 months. A total of 42% of migrants had their request of permit to stay in Italy rejected (Table 1).

### Clinical characteristics

Participants experienced a mean number of 4.8 (SD 2.7) traumatic events as measured by LEC (Table 2). The most frequent reported traumatic events were physical assault, captivity and severe human suffering. In terms of psychological distress, a total of 87 (34.5, 95% CI 28.7 to 40.7%) asylum seekers and refugees turned out to have clinically relevant symptoms; of these, 56 (64.4, 95% CI 53.4 to 74.4%) met the M.I.N.I. criteria for a psychiatric diagnosis, accounting for 22.2% of the whole sample. Post-Traumatic Stress Disorder (PTSD) and mood disorder were the most frequent diagnoses, accounting for 26 and 24% of those with clinically relevant psychological distress, respectively (Table 2). Table 2 also shows the proportion of participants with clinically relevant psychological distress with mild, moderate and severe depressive symptoms, respectively. The populations recruited in Rome and Verona were similar in terms of psychological distress, as shown by the proportion of asylum seekers and refugees with clinically relevant symptoms (35% in Verona vs 32% in Rome).

**Table 2:** Clinical characteristics in the total sample (*n* = 245) and in the sample of people with psychological distress (*n* = 87)

Variable	n	% or mean (SD)
**Traumatic events (LEC scale)**		
Mean number of traumatic events	250	4.8 (2.7)
**Psychological Distress**		
(GHQ-12 positive, cut off: ≥4)	87	34.5
Mean	252	2.8 (2.5)
**Diagnosis** (M.I.N.I. on GHQ-12 positive, n = 87)		
No diagnosis	31	35.6
Mood disorders	21	24.1
Anxiety	6	6.9
PTSD	23	26.4
Other	6	6.9
**Depressive symptoms severity** (HDRS on GHQ-12 positive, n = 87^a^)		
No depressive symptoms	37	44.0
Mild depressive symptoms	37	44.0
Moderate depressive symptoms	7	8.3
Severe depressive symptoms	3	3.6

*Abbreviations*: *SD* Standard deviation, *LEC* Life Events Checklist, *GHQ-12* 12-item General Health Questionnaire, *M.I.N.I*. Mini International Neuropsychiatric Interview; Hamilton Rating Scale for Depression (HRSD)

^a^missing data for 3

### Univariate association between psychological distress and LEC

**Fig. 1:**
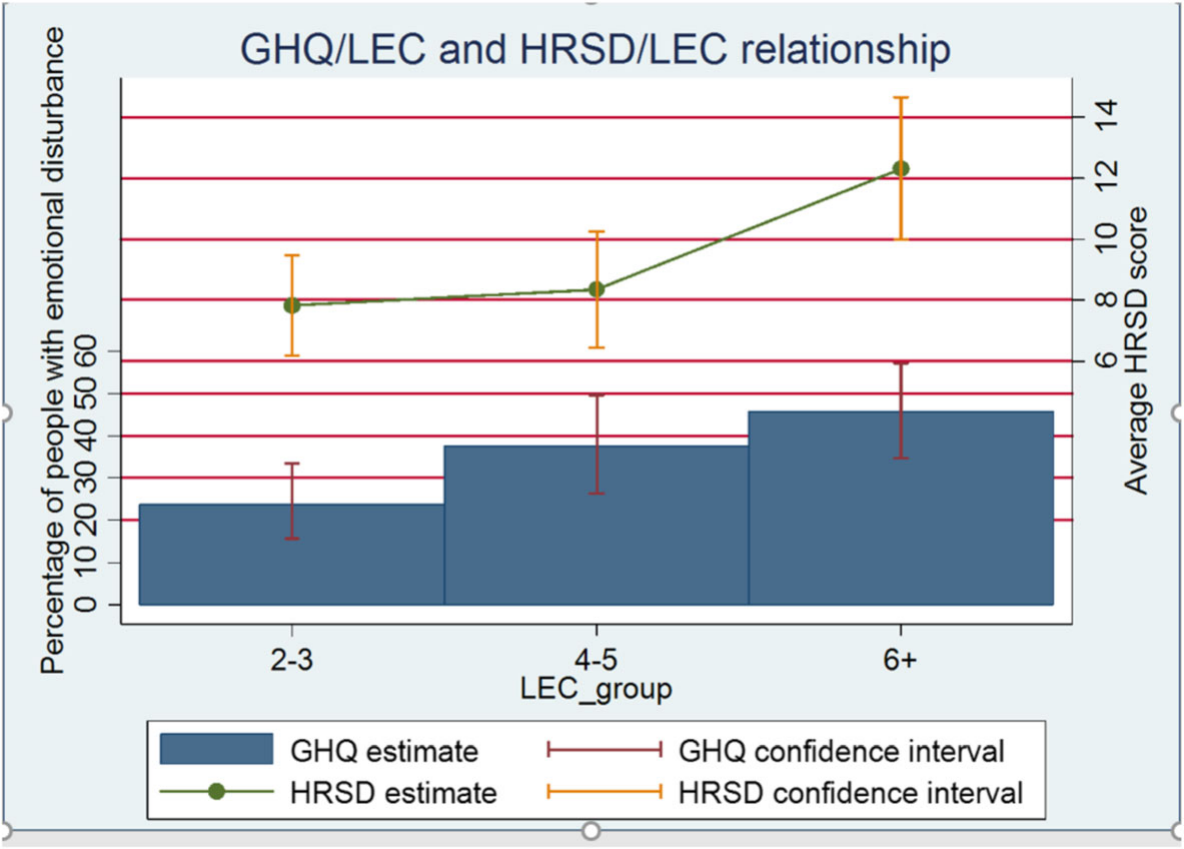
Psychological distress (percentage of GHQ positive) and depressive symptoms (average HRDS score) in relation with number of traumatic life events (LEC groups).GHQ: General Health Questionnaire; LEC: Life Events Checklist; HRDS: Hamilton Rating Scale for Depression

The distribution of asylum seekers with clinically significant psychological distress by number of traumatic events, represented in Fig. 1, highlighted a significant positive association (chi-square 9.50, *p*-value 0.002 for the trend test). Similarly, a positive association between depressive symptoms and number of traumatic events was highlighted (Jonckheere-Terpstra test, 2.722, *p*-value 0.006) (Fig. 1). Such results were confirmed by Spearman’s correlation on interval-level variables, with number of traumatic events positively associated with both the GHQ (rho = 0.148, *p*-value 0.019) and the HRSD (rho = 0.389, *p*-value < 0.001) scores.

### Regression analyses

Factors associated with psychological distress and depression are reported in Table 3. The sequential logit regression turned out to be globally statistically significant (*p*-value 0.002); looking at single regressions, significance was retained by the regression on GHQ-12 (*p*-value 0.003), while not on presence of a M.I.N.I. diagnosis (*p*-value 0.507). The regression on HRSD was significant as well (*p*-value < 0.001). Thus, results on single predictors were only analysed for the GHQ-12 and HRSD outcomes.

**Table 3:** Factors associated with psychological distress (GHQ-12 positive) in the total sample and with depression (HRSD score) in the sample of people with psychological distress

Explanatory variable	Psychological distress		Severity of Depression	
	***N*** = 245		***N*** = 82	
	Odds Ratio	95% CI	Coef.	95% CI
Educational level (at least high school level)	1.510	(0.829 t o2.751)	0.137	(−0.195 to 0.469)
Social support (good or very good vs other)	**0.409**	(0.225 to 0.744)	−0.082	(−0.419 to 0.254)
Permit to stay in Italy				
granted vs denied	**0.564**	(0.309 to 1.028)	0.212	(−0.137 to 0.560)
unclear vs denied	**2.428**	(0.649 to 9.085)	0.292	(−0.369 to 0.953)
Age (years)	1.039	(0.989 to 1.091)	0.007	(−0.019 to 0.033)
Marital status (married or cohabitant vs single)	0.781	(0.375 to 1.630)	−0.268	(−0.679; 0.144)
Time after departure (years)	0.919	(0.826 to 1.023)	−0.042	(−0.107 to 0.024)
Country of origin:				
Pakistan vs Africa	2.081	(0.967 to 4.479)	**−0.078**	(−0.486 to 0.331)
Other vs Africa	1.586	(0.621 to 4.049)	**−0.861**	(−1.394 to − 0.329)
Traumatic events (LEC) (number of events)	**1.213**	(1.072 to 1.372)	**0.176**	(0.111 to 0.244)

Significant parameter estimates (*p*-value < 0.05) marked in bold. In the case of variables with more than two categories, the global *p*-value was considered

Looking at each regression separately, the number of traumatic events turned out to be a risk factor for both GHQ-12 and HRSD. A good social support emerged as a protective factor towards GHQ-12, while migrants with unclear status were at higher risk of psychological distress than those holding or awaiting a permission.

As for the HRDS, having a country of origin outside Africa and different from Pakistan was, ceteris paribus, a strong protective factor.

Results of the sensitivity analyses (shown in Additional file) confirmed our findings in terms of statistical significance and direction of results, both in the case of the regression to predict psychological distress by using the Verona site only (Additional file, Table 1), and in the case of using HRSD as an ordinal variable (Additional file, Table 2).

## Discussion

The present study documented high rates of psychological distress and mental disorders amongst male asylum seekers and refugees recently resettled in Italy. About one-third showed clinically relevant psychological distress and one-fourth met the criteria for a psychiatric diagnosis, most often PTSD. Additionally, the number of traumatic events was found to be a risk factor for psychological distress and depressive symptoms.

These results expand previous findings that were limited to women or Syrian populations of asylum seekers and refugees [7–9]. As compared with women, men may be exposed to different trauma experiences, such as seeing someone being tortured by soldiers or having been imprisoned [18], and may also react differently to similar stressors. Similarly, there might be differences across different ethnic groups [19]. Our results would suggest that even though the type and psychological impact of traumatic events could be different between men and women of different background [10], their association with psychological distress, PTSD and depression seemed to be similar.

Our previous study [12] suggested that pre-migration, migration and post migration stressors and traumatic experiences are common in all individuals fleeing conflict or persecution, leaving their countries because their life and freedom are at risk. The present study identified a significant dose effect of traumatic experiences on mental health in recently resettled men across different backgrounds, whereby greater exposure to traumatic experiences was associated with higher severity of psychiatric symptoms, including PTSD. Further research could explore possible heterogeneity in such findings across ethnical groups and other sociodemographic variables.

In terms of time since trauma exposure, we found a positive association between psychological distress and mental disorders and pre-migration stressors [7], meaning that traumatic events experienced several months before resettlement still impact the psychological status of individuals. This might be related to the characteristics of the traumatic events experienced by individuals, that include for example witnessing or receiving physical violence, witnessing people being killed, witnessing bombings or mass killings or massacres, having been tortured, and having been recruited by rebels [19]. At the same time, our results highlighted a positive association also between psychological distress and post migration factors. Even though post-migration stressors have not been taken into account in the majority of studies, recent data highlighted that, during the post-migration phase, refugees may experience different types of difficulties that can hamper recovery, increase mental ill health or be instrumental for the development of mental health ill [9]. In the present study we found that post-migration stress seems to be a subtler but persistent character as compared with previously experienced traumatic events, which tend to be sudden and traumatic. Post-migration stress and mental ill health are in the other hand likely to be reciprocally associate in the resettlement phase: it could be explained both for the effect of post-migration traumatic events and for the impact of traumatic events occurred before migration and during initial migration phases that could make refugees particularly vulnerable to psychological suffering even after resettlement [9, 18].

Giacco, who recently critically analysed the literature on this topic, identified three different time points in the post migration phase, each of them with a relevant association with mental health: initial settlement in the host country, integration in the host country and challenges to or revocation of the immigration status [6]. A longer duration of asylum-seeking process and lack of information can generate prolonged stress, such as social isolation, discrimination and victimisation. Moreover, data from literature confirmed that “bridging social networks” are an important protective factor [7]. Our study corroborated the role of post migration factors in predicting psychological distress and depression, underlining the crucial role of social support and procedures related to permission and immigration status.

This study has important limitations. First, it may be difficult to generalize results in view of the specific characteristics of the population included and of the migration and resettlement context; similarly, given that only men were included, we do not know if findings may also apply to women, who usually show higher rates of common mental health conditions. There are additional limitations related to the study design. A logistic regression analysis was employed in order to test the possible association of a number of independent variables with the occurrence of psychological distress. However, the cross-sectional nature of this study cannot establish a cause-effect relationship, and therefore all significant associations must be considered hypothesis-generating rather than hypothesis-testing.

Despite these limitations, there are a number of important clinical, research and policy implications. The association between the amount of traumatic events and mental health conditions suggests the need of developing services to assist refugees and asylum seekers to address the multi-faceted problems they experience, implementing holistic through assistance with problems such as social support in host country, legal problems concerning permit status and asylum procedure, and family reunification, as well as addressing trauma and mental health issues. Future research should attempt to better understand the risk and resilience factors that may be related to the mental health of refugees and asylum seekers and their overall well-being, adaptation and quality of life following arrival in host countries. Studies investigating individuals at different stages of the asylum-seeking process, in addition to longitudinal studies, may assist in identifying important post-migration stressors. A second implication is that the consistent association between psychopathology and post-migration problems found in this study, and reported in other populations of refugees and asylum seekers, suggests the need for an asylum system that aims to reduce post-migration stress and increase support [20]. At the moment, however, there are concerns related to the decision-making process of some asylum applications, and to the well-documented difficulties for asylum seekers and refugees in accessing social and health care support. These factors may therefore contribute to increase rather than mitigate psychological stress. There is an urgent need to implement policies that may reduce post-migration problems and assist adaptation and integration.

## Conclusion

Overall, considering the findings of this study and the substantial proportion of refugees and asylum seekers identified as having mental health difficulties by epidemiological studies, it may be of particular importance to implement approaches for early identification and support. The inclusion of a mental health component in the overall provision of care for refugees would represent a feasible and sustainable way to reduce the burden of psychological distress and to prevent the development of mental health conditions. As effective psychosocial interventions are available for asylum seekers and refugees with PTSD and/or other mental health conditions [21–23], recognition of psychological distress would reduce stigma, aid in diagnosis and increase access to specialized mental health care. This would prevent a deterioration of serious mental health conditions, reduce emotional distress, contribute to better adjustment and reduce the burden of assimilation on hosting countries, as also recently pointed out by the UCL–Lancet Commission on Migration and Health [24].

## Supplementary Information

**Additional file 1: Table 1.** Factors associated with psychological distress (GHQ-12 positive) in the sample recruited in the Verona site. **Table 2.** Factors associated with depression (HRSD group score) in the sample of people with psychological distress.

## Data Availability

The datasets obtained and/or analysed during the current study are available from the corresponding author on reasonable request.

## Abbreviations

PTSD: Post Traumatic Stress Disorder
SPRAR system: Protection system for asylum seekers and refugees
LEC: The Life Events Checklist
CAPS: Clinician Administered PTSD Scale
HRSD: Hamilton Rating Scale for Depression
GHQ-12: General Health Questionnaire-12 items
M.I.N.I.: MINI-International Neuropsychiatric Interview
SD: Standard deviation
CI: Confidence interval
