# Supplementary material for: Association of traumatic events with levels of psychological distress and depressive symptoms in male asylum seekers and refugees resettled in Italy
Source: BMC Psychiatry. 2020 Dec 1;20:576. doi: 10.1186/s12888-020-02988-0 (PMC7709257; doi:10.1186/s12888-020-02988-0)
Supplement: Supplementary file 1 — Additional file 1: Table 1. Factors associated with psychological distress (GHQ-12 positive) in the sample recruited in the Verona site. Table 2. Factors associated with depression (HRSD group score) in the sample of people with psychological distress. [file 12888_2020_2988_MOESM1_ESM.docx]

**Additional file : SENSITIVITY ANALYSIS**

**Tab 1. Factors associated with psychological distress (GHQ-12 positive) in the sample recruited in the Verona site**

|  | **Psychological distress** | |
| --- | --- | --- |
| **Explanatory variable** | **N = 197** | |
|  | **Odds Ratio** | **95% CI** |
| Educational level (at least high level) | 1.975 | (0.967-4.034) |
| Social support  (good or very good vs other) | **0.360** | (0.173-0.746) |
| Permit to stay in Italy  granted vs denied  unclear vs denied | 0.513  3.497 | (0.254-1.036)  (0.800-15.285) |
| Age (years) | 1.037 | (0.981-1.096) |
| Marital status (married or cohabitant vs single) | 0.830 | (0.345-1.995) |
| Time after departure (years) | 0.892 | (0.789-1.008) |
| Country of origin:  Pakistan vs Africa | .1.342 | (0.544-3.308) |
| Other vs Africa | 1.025 | (0.326-3.225) |
| Traumatic events (LEC) (number of events) | **1.733** | (1.377-2.182) |

**Significant parameter estimates (p-value <0.05) marked in bold. In the case of variables with more than two categories, the global p-value was considered**

**Tab 2. Factors associated with depression (HRSD group score) in the sample of people with psychological distress**

|  | **Severity of Depression** | |
| --- | --- | --- |
| **Explanatory variable** | **N = 82** | |
|  | **Odds Ratio** | **95% CI** |
| Educational level (at least high level) | 1.187 | (0.442-3.190) |
| Social support  (good or very good vs other) | 1.381 | (0.510-3.739) |
| Permit to stay in Italy  granted vs denied  unclear vs denied | 1.849  1.774 | (0.644-5.308)  (0.224-14.021) |
| Age (years) | 1.033 | (0.956-1.117) |
| Marital status (married or cohabitant vs single) | 0.305 | (0.085-1.092) |
| Time after departure (years) | 0.871 | (0.706-1.074) |
| Country of origin:  Pakistan vs Africa | **0.730** | (0.230-2.313) |
| Other vs Africa | **0.087** | (0.014-0.527) |
| Traumatic events (LEC) (number of events) | **1.548** | (1.248-1.920) |

**Significant parameter estimates (p-value <0.05) marked in bold. In the case of variables with more than two categories, the global p-value was considered**
